# Supplementary material for: Characterizing Interdisciplinarity of Researchers and Research Topics Using Web Search Engines
Source: PLoS One. 2012 Jun 13;7(6):e38747. doi: 10.1371/journal.pone.0038747 (PMC3374816; doi:10.1371/journal.pone.0038747)
Supplement: Appendix S1 — Detailed methods of web search engine-based data collection used in this study. (DOC) [file pone.0038747.s001.doc]

**Appendix S1**

To create lists of research topics and researchers’ names, we had several student volunteers exhaustively explore the following conference websites manually and collect keywords or phrases related to scientific research as well as names of all researchers involved, including organizers, committee members, session chairs, presenters, authors/coauthors, and panelists. Each conference website was explored by at least two independent students. These conferences were chosen primarily because of our own familiarity with them (which was necessary in order to check and manually correct the raw data coming from the student volunteers).

*Mathematical Biology:*

SMB 2006 http://www.siam.org/meetings/ls06/

SMB 2007 http://abacus.bates.edu/~mgreer/smb_jsmb_2007/

SMB 2008 http://www.fields.utoronto.ca/programs/scientific/CMM/

08-09/SMB/index.html

SMB 2009 http://www.math.ubc.ca/Research/MathBio/SMB2009/

*Artificial Life:*

ALIFE X (2006) http://www.alifex.org/

ECAL 2007 http://www.ecal2007.org/

ALIFE XI (2008) http://alifexi.alife.org/

ECAL 2009 http://www.ecal2009.org/

*Network Science:*

NetSci 2006 http://vw.indiana.edu/netsci06/

NetSci 2007 http://www.nd.edu/~netsci/

NetSci 2008 http://www.ifr.ac.uk/netsci08/

NetSci 2009 http://www.netsci09.net/

*Bio-inspired Information Technology:*

BIONETICS 2006 http://www.bionetics.org/2006/

BIONETICS 2007 http://www.bionetics.org/2007/

BIONETICS 2008 http://www.bionetics.org/2008/

BIONETICS 2009 http://www.bionetics.org/2009/

The keywords and phrases related to scientific research collected by the students were manually edited and compiled by the authors into the following list of 40 research topics used in this study. They were sorted into four major categories and the numbers of words were made similar across those categories in order to maintain a good balance among different disciplines.

[Biological and medical sciences, 13 words]

*biology*

*cancer*

*cell*

*disease*

*ecology*

*ecosystem*

*evolution*

*gene*

*immunology*

*molecule*

*neuron*

*physiology*

*protein*

[Physical sciences, 9 words]

*chemistry*

*complex network*

*complex system*

*dynamics*

*emergence*

*mathematics*

*modeling*

*physics*

*simulation*

[Engineering and robotics, 9 words]

*algorithm*

*application*

*cognition*

*computer*

*control*

*engineering*

*motor*

*robot*

*software*

[General terms, 9 words]

*communication*

*economy*

*information*

*intelligence*

*network*

*science*

*signal*

*social network*

*system*

In collecting researchers’ names, the student volunteers were instructed to omit middle initials and replace any letters with diacritics (e.g., accents, umlauts, etc.) by normal alphabet letters without diacritics. These rules were implemented so as to make data inspection and correction easier. The collected lists of researchers’ names were then aggregated into a single text file and carefully examined by the authors to correct any recognizable mistakes (misspellings, duplications, etc.). As a result, we had over 4,050 names collected in total.

To conduct systematic web searches using Google, we wrote a simple program code in Java that sequentially searches for each pair of keywords stored in a plain text file and then extracts and records their relatedness (i.e., number of search hits) from the search result. The code was developed using with Google Web Search API just for internal use for this study and not for public release, but it is available from the authors upon request.

The original list of researchers’ names was too long for our data collection (i.e., the number of pairs, 4,050 choose 2, was beyond eight million), so we first conducted systematic web searches for each researcher’s name only (together with the filtering word “research”). Then we sorted the results in terms of search hits and selected the top 1,000 researchers for the main data collection and analysis.

For the main data collection, each search query (in which “research” was always included) was searched three times at different times in a day in order to improve the reliability of search results. A known problem in using Google search results is that it sometimes returns an orders of magnitude larger (or smaller) number as search hits, which would strongly influence if the results were averaged as is. To avoid such errors, we discarded either the largest or smallest result, whichever was farther away from the median result, before calculating a mean. If both were equally distant from the median, none of them was discarded, i.e., the mean became the median.

We used programming language Python and its NetworkX module [31] for network visualization and analysis, and also Wolfram Research’s Mathematica for statistical analysis. Codes for these analyses were also developed for internal use only, but can be shared upon request.
